# Catamenial Hematuria in Mixed Gonadal Dysgenesis (45,X/46,XY): A Case Report of a Rare Presentation

**DOI:** 10.7759/cureus.109688

**Published:** 2026-05-26

**Authors:** Abirami Anbarasu, Jegadeesh Sundaram, Prakash Agarwal, Kartthick Subburaj, Bathrenathh Balasubramanian

**Affiliations:** 1 General Surgery, Sri Ramachandra Institute of Higher Education and Research, Chennai, IND; 2 Pediatric Surgery, Sri Ramachandra Institute of Higher Education and Research, Chennai, IND; 3 Pediatric Surgery, Apollo Hospitals, Chennai, IND; 4 Pediatric Surgery, Sri Ramachandra Institute of Higher Education and Research, Chennai, Tamil Nadu, IND; 5 Radiology, Sri Ramachandra Institute of Higher Education and Research, Chennai, IND

**Keywords:** cyclical hematuria, minimally invasive surgery, mixed gonadal dysgenesis, persistent müllerian duct syndrome (pmds), turner mosaicism

## Abstract

Mixed gonadal dysgenesis (MGD) is a rare disorder of sex development (DSD) associated with 45,X/46,XY mosaicism and variable phenotypic presentation. Mullerian remnants may persist but are typically non-functional. We report a 16-year-old phenotypic male with hypospadias and a unilateral undescended testis with prior resection of a streak gonad containing fallopian tube structures, who presented with cyclical hematuria and lower abdominal pain. Imaging suggested a Müllerian duct cyst. Diagnostic laparoscopy and cystoscopy revealed a cystic structure communicating with the posterior urethra, initially mimicking a diverticulum. Surgical excision was performed, and histopathology demonstrated uterine tissue with proliferative endometrium. The symptoms were attributed to catamenial bleeding from functional Müllerian tissue. This case highlights a rare presentation of MGD and emphasizes the importance of considering Müllerian remnants in phenotypic males with cyclical hematuria.

## Introduction

The Müllerian duct is the embryological structure from which the female reproductive organs develop. The Wolffian duct is the precursor structure to male internal genitalia organ development. Pathology related to the growth of these structures can lead to disorders of sexual development. The four major groups of disorder of sex development (DSD) patients are: female pseudohermaphroditism (46,XX DSD), male pseudohermaphroditism (46,XY DSD), mixed gonadal dysgenesis (MGD), and true hermaphroditism [1]. It is estimated that one in 4,500 babies is born with DSD globally [2]. MGD is a rare DSD characterized by asymmetrical gonadal growth, typically presenting with one streak gonad and one dysgenetic testis. MGD has an incidence of 1 in 15,000 to 1 in 30,000 live births. MGD commonly occurs in association with 45,X/46,XY mosaicism and demonstrates variable gonadal differentiation between the two sides of the body. Incomplete regression of Müllerian derivatives can occur secondary to deficient anti-Müllerian hormone (AMH) secretion or impaired receptor responsiveness [3]. Although these remnants are usually rudimentary and asymptomatic, they may occasionally present with clinical manifestations [4]. Patients with dysgenetic gonads carry a recognized long-term risk of gonadal malignancy, particularly gonadoblastoma. We report a rare case of MGD presenting as cyclical hematuria due to functional Müllerian tissue communicating with the urinary bladder, which was managed by minimal access surgery.

## Case presentation

History of presenting illness

A 16-year-old individual reared as a male presented with complaints of cyclical lower abdominal pain and hematuria occurring for about three to four days each month over the preceding few months. There was no history of trauma, fever, or voiding difficulty.

History

The patient had a history of hypospadias, for which surgical repair was performed at 1 year of age. Clinical suspicion of unilateral left-sided cryptorchidism warranted investigation, and hence, diagnostic laparoscopy performed at 1 year of age revealed a streak gonad on the left side, which was excised. Histopathological examination of the streak gonad demonstrated it to be a rudimentary fallopian tube. Subsequent karyotyping at 1 year of age revealed mosaicism (45,X/46,XY). No further investigations or follow-up were done following this.

Clinical Examination

On present-day examination, the patient had short stature (145 cm) with widely spaced nipples and a mildly webbed neck, suggestive of Turner phenotype. Local examination of the external genitalia revealed male phenotype with normal phallic size. The right testis was present within the scrotum, while the left hemiscrotum was empty.

Per-abdominal examination revealed a soft abdomen with tenderness in the hypogastrium, without guarding or rigidity.

Investigations

Laboratory evaluation showed a hormonal panel within normal limits (Table 1). 

**Table 1:** Serum hormonal workup showing testosterone, LH, FSH, estradiol, and AMH within normal limits. LH, luteinizing hormone; FSH, follicle-stimulating hormone; AMH, anti-Müllerian hormone; IU, international units; mIU, milli-international units

Hormone	Serum level	Reference range for 16-year-old boys
Testosterone	619 ng/dL	264-916 ng/dL
LH	3.2 IU/L	1.24-8.6 IU/L	
FSH	1.8 mIU/mL	1.5-12.4 mIU/mL
Estradiol	15 pg/mL	10-40 pg/mL
AMH	13 ng/mL	<128 ng/mL

Normal hormonal panel suggested that the patient had target organ hormone resistance rather than an abnormality of hormone-secreting organs.

Imaging

**Figure 1:**
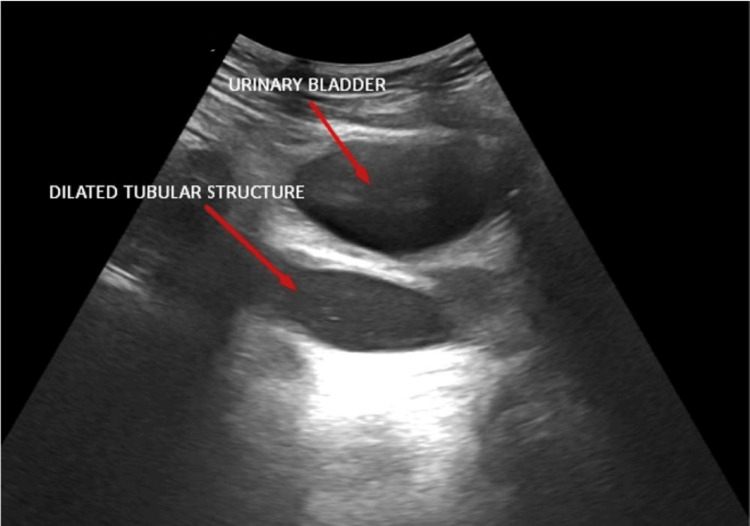
Dilated, fluid-filled tubular structure adjacent to the urinary bladder.

The patient was evaluated further by imaging. Ultrasonography of the abdomen demonstrated a dilated, tubular, fluid-filled cystic structure of size 5.0 x 1.9 cm in the midline pelvis, suggestive of a Müllerian duct cyst (Figure 1). Further information could not be obtained from the ultrasound; hence, a diagnostic laparoscopy was planned to directly visualize the pelvic pathology.

Intervention

**Figure 2:**
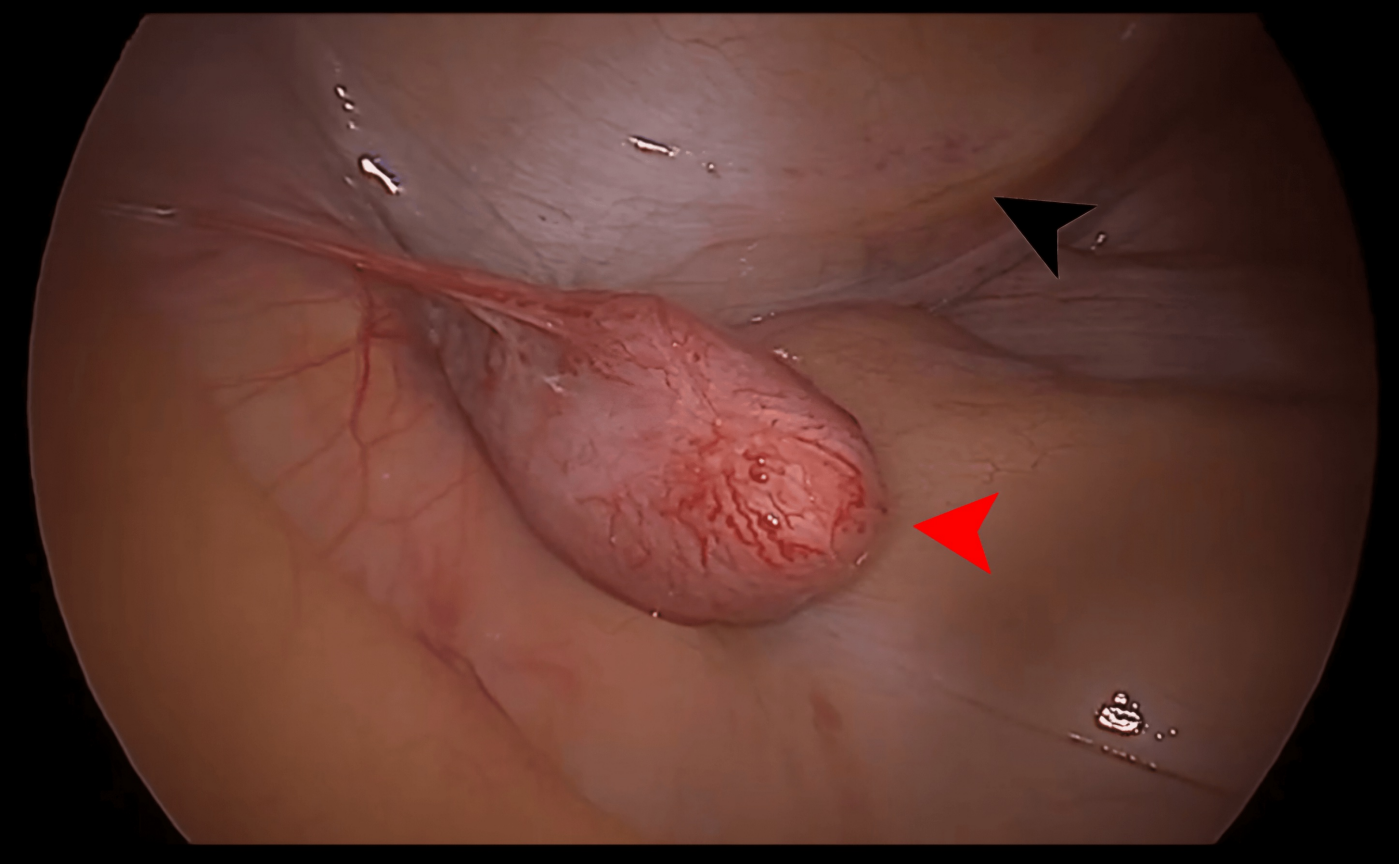
Laparoscopic view showing a cystic structure adjacent to the urinary bladder, initially suspected to be a bladder diverticulum. Red arrowhead: cystic structure. Black arrowhead: urinary bladder.

**Figure 3:**
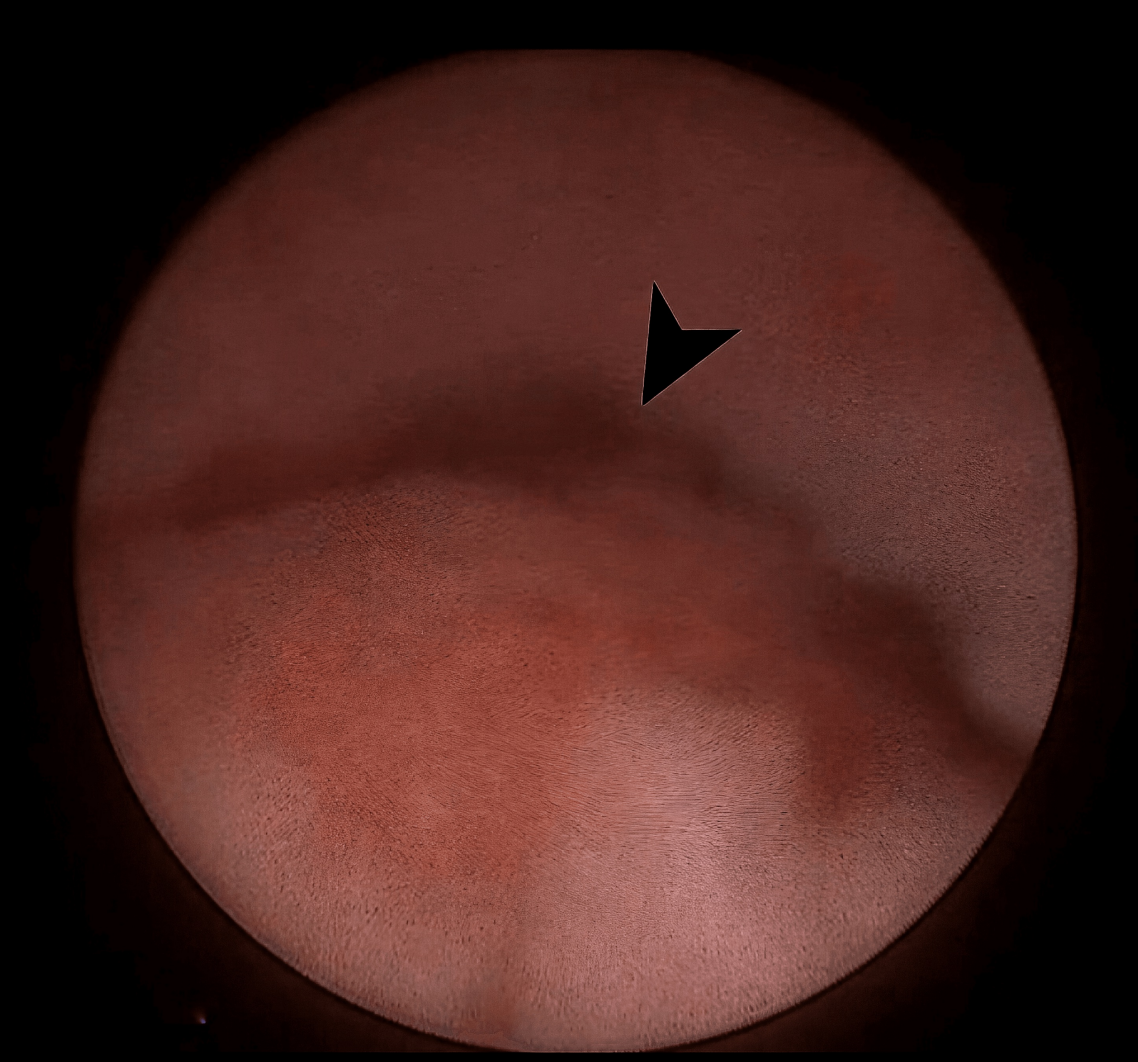
Cystoscopic view demonstrating a fistulous opening in the urethra adjacent to the prostatic utricle. Black arrowhead: fistulous opening.

Diagnostic laparoscopy revealed a cystic structure adjacent to and abutting the urinary bladder, resembling a bladder diverticulum (Figure 2). Cystoscopy showed a wide orifice of the prostatic utricle (Figure 3), which could be negotiated with the scope, revealing a blind ending cavitatory structure with luminal mucus and hyperemic lining.

**Figure 4:**
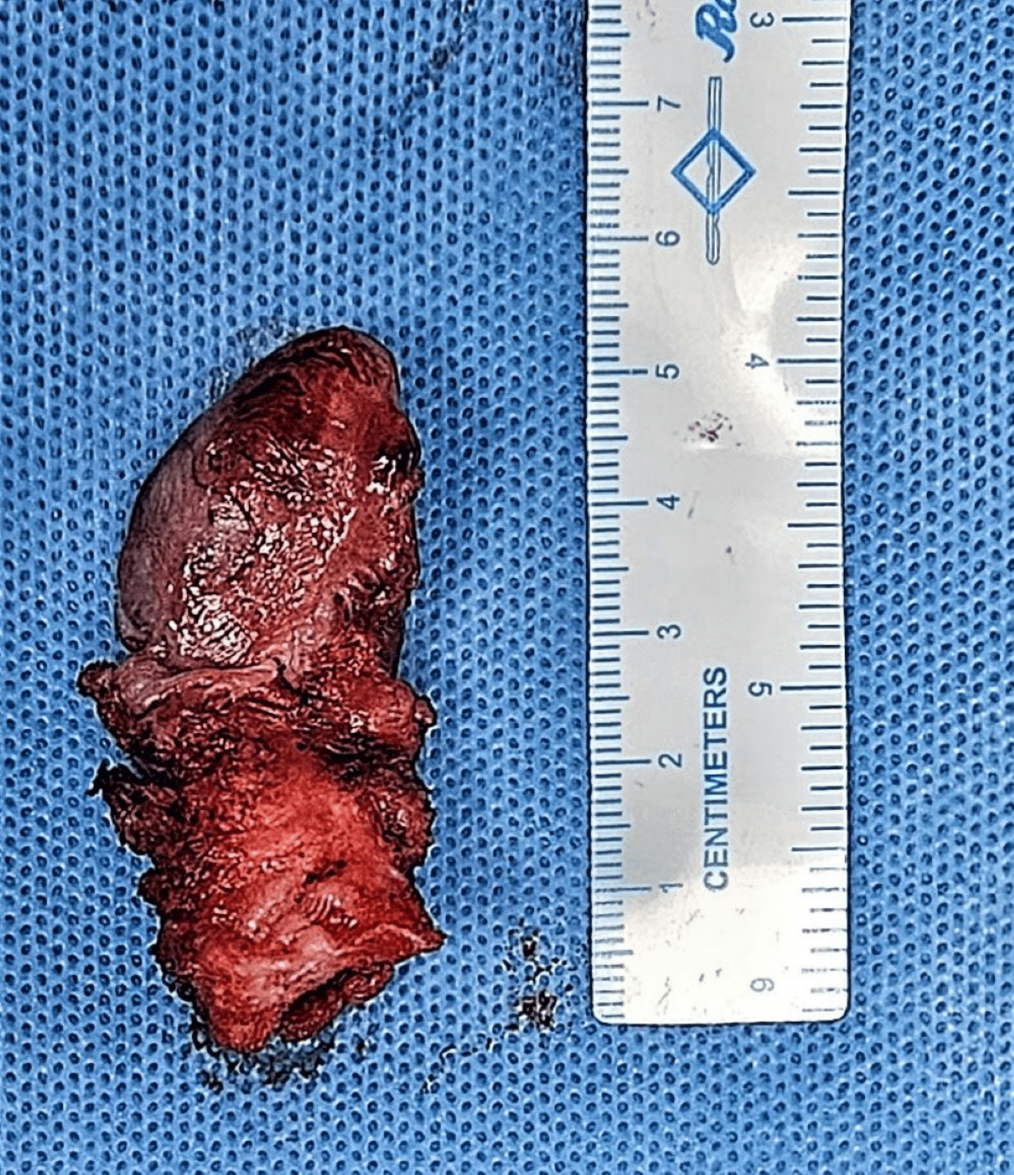
Gross specimen following excision, oriented parallel to the scale.

Laparoscopy served both a diagnostic and therapeutic role, wherein the cystic diverticulum was surgically excised and its attachment to the posterior urethra was sutured. The excised specimen measured 5 × 2 × 2 cm, as shown in Figure 4. The postoperative period was uneventful, with no recurrence of symptoms at eight-month follow-up.

Histopathology

**Figure 5:**
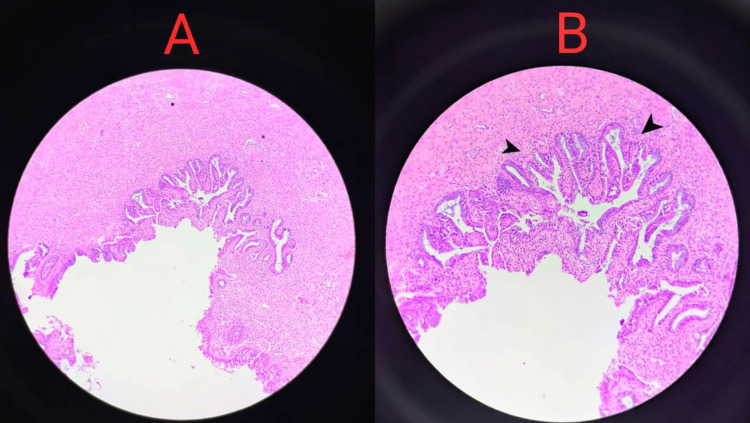
(A, B) Hematoxylin and eosin-stained tissue at ×10 and ×20 magnification showing cervical tissue with chronic papillary endocervicitis and endocervical glands. Arrowheads: endocervical glands.

**Figure 6:**
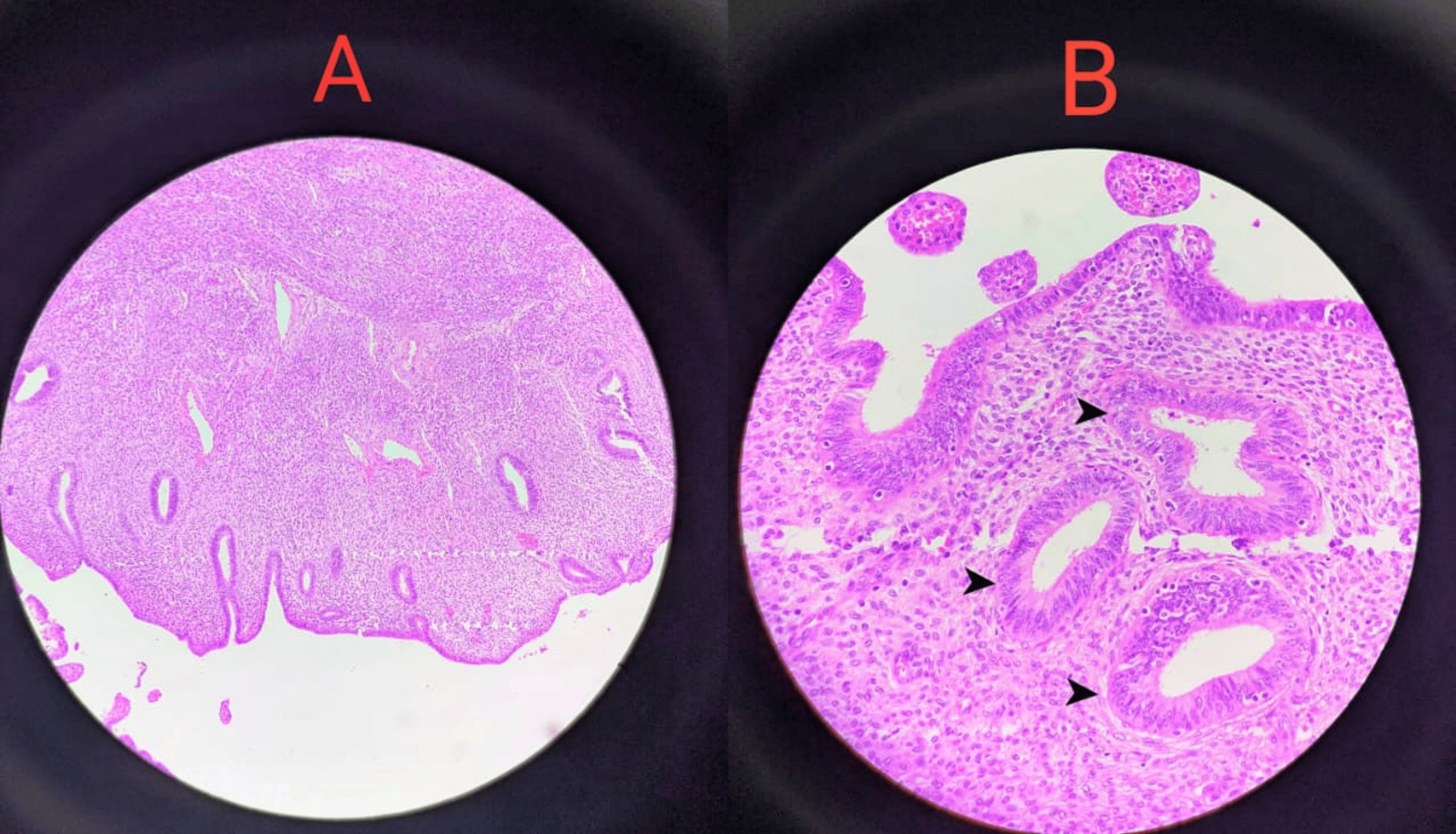
(A, B) Hematoxylin and eosin-stained tissue at ×10 and ×40 magnification showing uterine endometrium in the proliferative phase with endometrial glands (arrowheads) and myometrium composed of smooth muscle bundles. Arrowheads: endometrial glands.

Histopathological examination revealed uterine tissue containing both cervical tissue showing chronic papillary endocervicitis (Figures 5A-5B) and proliferative endometrium (Figures 6A-6B), confirming the presence of functional Müllerian structures.

Final Diagnosis

The final diagnosis was Persistent Müllerian Duct Syndrome (PMDS) in a case of MGD in a phenotypic male.

The cyclical hematuria was attributed to catamenial bleeding from ectopic uterine tissue communicating with the posterior urethra in the setting of MGD.

## Discussion

Gonadal dysgenesis refers to the abnormal or incomplete development of the gonads due to disruptions in germ cell migration or improper organization within the fetal gonadal ridge. This condition can arise from numerical or structural abnormalities of the sex chromosomes, as well as mutations in genes responsible for urogenital ridge development and the sex differentiation of the bipotential gonad, including GATA4, WT1 [5]. MGD is one of the types in which karyotyping shows 45,X/46,XY, and clinical features reveal asymmetric gonadal differentiation and sometimes persistence of Müllerian duct derivatives due to impaired AMH function [6]. PMDS may coexist with MGD, though rare, and when it does, it usually leads to complex anatomical and clinical presentations, and hence the need to tailor management is required [7]. PMDS generally presents in phenotypic males with normal virilization; however, literature shows that it is sometimes a rare presentation among MGD cases as well. Cyclical hematuria suggests that hormonally responsive Müllerian tissue containing functional endometrium is present, which undergoes periodic shedding [8]. Such presentations are exceedingly rare since most reported cases of Müllerian remnants in MGD present with incidental findings, infertility, inguinal hernia, or pelvic masses.

During male fetal development, Sertoli cell-derived AMH normally induces regression of Müllerian structures [9,10]. In patients with MGD, inadequate hormone production or defective Müllerian responsiveness may permit persistence of uterine or tubal derivatives. Although these remnants are frequently asymptomatic in childhood, hormonally responsive endometrial tissue may become clinically active after puberty. This explains the delayed onset of symptoms in our patient, who developed cyclical hematuria only during adolescence.

A valuable learning point from this case is the clinical consequences of incomplete excision of Müllerian structures during initial surgical management [11]. In patients with MGD, early surgical interventions are directed toward the removal of dysgenetic gonads due to malignancy risk and correction of external genital anomalies such as hypospadias. However, asymptomatic or difficult-to-access Müllerian remnants may be left in situ. As demonstrated in this case, retained Müllerian tissue has the potential to present later in life with complications including infection, cyst formation, compressive symptoms, or hormonally mediated bleeding. The psychosocial burden of diagnosis and treatment in older children, particularly in patients presenting with atypical symptoms or undergoing procedures, should also be carefully considered [12]. Ravirajendran et al. reported a similar case of Müllerian remnants presenting as a pelvic cyst in a young adult with MGD, emphasizing the long-term sequelae of retained structures [13].

Another notable highlight is the role played by minimally invasive surgery (MIS) in the management of such cases. Laparoscopic excision offers superior visualization of deep pelvic anatomy, aiding meticulous dissection while preserving vital structures such as the bladder, ureters, and vas deferens [14]. In our case, the availability of combined laparoscopic, cystoscopic, and histopathological correlation strengthens diagnostic accuracy and highlights the importance of multimodal evaluation. In patients with prior abdominopelvic surgeries, adhesions and distorted anatomy can increase operative complexity; however, MIS remains advantageous in reducing postoperative pain, hospital stay, and morbidity. In our case, successful laparoscopic excision of the Müllerian remnant resulted in complete resolution of symptoms, demonstrating the feasibility and effectiveness of MIS in experienced hands.

Smith-Harrison et al. described a similar case in 2015 of a 27-year-old male presenting with painless hematuria and hematospermia; examination showed bilateral descended testes and a normal penis. MR urogram revealed a right unicornuate uterus with an endocervical canal terminating in the right seminal vesicle, and karyotyping showed a 46,XY karyotype. Full hormonal panel - AMH, estradiol, follicle-stimulating hormone (FSH), luteinizing hormone (LH), and testosterone - was within normal limits. Cystoscopy showed a pinpoint orifice at the level of the verumontanum communicating with the uterus. The patient was managed with robotic excision of the Müllerian remnant [15].

The major differences between our case and the Smith-Harrison et al. case are compared in Table 2.

**Table 2:** Major differences between our index case and Smith-Harrison et al. case. Data from Smith-Harrison et al. [15].

	Index case	Smith-Harrison et al. case
Age at presentation	16 years	27 years
Complaint	Cyclical hematuria associated with lower abdominal pain	Painless hematuria and hematospermia
Examination	Right testis present, left testis absent	Bilateral testis palpable
Imaging	USG showed a tubular fluid-filled cyst adjacent to the urinary bladder on the left side	MR urogram showed a right-sided unicornuate uterus in connection with the seminal vesicles.
Cystoscopy	Scope negotiable orifice of the prostatic utricle leading to a cystic structure	Pinpoint orifice at the level of the verumontanum.
Management	Laparoscopic excision	Robotic excision

This present case is noteworthy due to the rare presentation of cyclical hematuria in an adolescent with MGD and persistent Müllerian remnants, which was further managed using minimally invasive techniques. Very few reports in the English literature describe cyclical hematuria secondary to functional Müllerian tissue, and none include a patient with MGD. This patient was managed using minimally invasive surgery, which is even more rarely documented and may represent one of the first such cases in the English-language literature, thereby highlighting the uniqueness of this case.

Long-term follow-up in patients with MGD, especially clinical surveillance through puberty, is a necessity, as hormone-induced clinical features of residual embryological structures may present during this period. A multidisciplinary team comprising pediatric urologists, endocrinologists, radiologists, surgeons, genetic counselors, and psychotherapists is essential to ensure timely diagnosis and optimal management of such rare presentations.

## Conclusions

This case highlights a rare and clinically significant presentation of MGD with PMDS manifesting as cyclical hematuria. It emphasizes the importance of early recognition of persistent Müllerian structures, advocates for complete surgical excision when feasible, and demonstrates the effectiveness of minimally invasive techniques in the management of complex pelvic pathology. Our case report contributes to the limited literature available on symptomatic Müllerian remnants in DSD and underscores the need for vigilance in long-term follow-up.

Preoperative imaging and intraoperative assessment also hold important roles and should aim to delineate the extent of Müllerian remnant structures, especially in known DSD patients. A proactive surgical approach, preferably at a younger age, may help prevent delayed complications, avoid negative psychosocial impacts and reduce the need for re-intervention in adolescence or adulthood.
